# Investigation of the Properties of Linen Fibers and Dressings

**DOI:** 10.3390/ijms231810480

**Published:** 2022-09-09

**Authors:** Tomasz Gębarowski, Izabela Jęśkowiak, Benita Wiatrak

**Affiliations:** 1 Department of Biostructure and Animal Physiology, The Wroclaw University of Environmental and Life Sciences, Kożuchowska 1/3, 51-631 Wroclaw, Poland; 2 Department of Pharmacology, Faculty of Medicine, Wroclaw Medical University, Mikulicza-Radeckiego 2, 50-345 Wroclaw, Poland

**Keywords:** flax, fiber, fibroblast

## Abstract

In antiquity, flax was used as a dressing for healing wounds. Currently, work is underway on the genetic modification of flax fibers to improve their properties. Genetic modifications have resulted in an increased content of antioxidants and more favorable mechanical properties. The works published so far have presented independent tests of fibers and dressings after appropriate technological treatments in cell cultures. This study aimed to compare the properties of the fibers and the dressing produced in cell cultures—hamster fibroblasts—V79. The research material was traditional NIKE fibers; genetically modified M, B, and MB fibers; and linen dressings obtained from these fibers. The extract from 48-h incubation of 40 mg of fiber in the culture medium, which was desolved into 10, 20, and 30 mg, was administered to the cell culture. On the other hand, a linen dressing was placed on cells with an area of 0.5 cm^2^, 1 cm^2^, 1.5 cm^2^, and 2 cm^2^. Cells with fiber or dressing were incubated for 48 h, and then, biological tests were performed, including cell viability (in propidium iodide staining), cell proliferation (in the SRB assay), evaluation of the intracellular free radical level (in the DCF-DA assay), genotoxicity (in the comet assay), assessment of the apoptotic and necrotic cells (in staining anexin-V and iodide propidium), the course of the cell cycle, and the scratch test. The correlation between apoptosis and genotoxicity and the levels of free radicals and genotoxicity were determined for the tested linen fibers and fabrics. The tests presented that the fibers are characterized by the ability to eliminate damaged cells in the elimination phase. However, the obtained fabrics gain different properties during the technological processing of the fibers into linen dressings. Linen fabrics have better regenerative properties for cells than fibers. The linseed dressing made of MB fiber has the most favorable regenerative properties.

## 1. Introduction

Serious skin wounds caused by injuries, burns, or diabetes predispose patients to severe disability and even death. In addition, wound healing may slow down during pathological conditions or aging, and wounds may become chronic [1]. Traditional wound dressings (e.g., cotton bandages and gauze) are the first type of commonly used wound dressings [2]. However, chronic wounds are insufficient to ensure effective healing and can significantly delay wound closure [3].

Solutions are sought among linen products. Common flax (Latin Linum usitatissimum) is a plant that has been used for thousands of years. The health benefits of flax seeds are mainly attributed to the omega-3 fatty acids and fiber they contain. It should not be forgotten that flax is also a valuable source of antioxidant ligands that are widespread in various parts of plants as glycoside conjugates associated with a fibrous plant component. Lignans prevent the toxic effects of oxidation by sequestering free radicals. This process involves inhibiting the attack of lipid peroxidation. In addition, phenolic compounds stimulate the synthesis of collagen, one of the main components of the skin’s connective tissue [4]. Flax fibers also contain many active substances, such as unsaturated fatty acids, 4-hydroxybenzoic acid, sterols (campesterol and β-sitosterol), ferulic acid, and polyhydroxy butyrate (PHB). Cannabidiol (CBD), contained in fibers, exerts an analgesic effect. Flax fibers are characterized by two very important properties, such as the ability to sweep up moisture, which is used for oozing wounds, and being hypoallergenic—thanks to which, a dressing made of such material is also available to people suffering from an overactive immune system manifested by a tendency toward allergic reactions [5].

Processed flax fibers are characterized by good mechanical properties, low density, and tensile strength from 264 to 2000 MPa [6,7,8,9,10]. Obtaining transgenic flax varieties improves the mechanical properties of Linum raw materials and increases the wound-healing capacity of fabrics obtained from flax fibers with more favorable properties [3,6]. In addition, the resulting flax fiber dressings are fully biodegradable.

A narrow team of researchers researched flax fabrics in cell cultures and humans. The most important conclusions from the research conducted so far are presented in Table 1.

In this study, we prepared flax fibers and fabrics based on genetically modified flax fibers to evaluate their properties using Chinese Hamster Lung (V79) fibroblast cells, commonly used to test DNA damage, mutagenicity, and toxicity. V79 cells are immortal, easily mutagenized, and have a shortened cell cycles [18,19]. The V79 lineage can also be used to detect structural and numerical chromosomal damage by measuring the micronucleus formation in mitotic-divided interphase cells. In addition, V79 is well-established in toxicology studies. The stability of the karyotype and morphology makes them suitable for gene toxicity tests with low background aberrations. Ford and Yerganian developed this cell line in 1958 from the lung tissue of a young male Chinese hamster. 

The study aimed to compare the properties of traditional flax fibers and NIKE dressings with the M, B, and MB-modified flax in the V79 model line. 

## 2. Results

### 2.1. Cell Viability

The study compared the cell survival of V79 cultures after 24-h incubation at 37 °C of four types of flax fibers and NIKE, M, B, and MB fabrics. Increased cell viability was observed after incubation with flax fabrics compared to flax fibers. The highest viability values of the cultured V79 cells were observed after incubation with linen fabric, depending on its tested fabric size. The highest viability of V79 cells for the fabric 0.5 cm was observed for the M fabric (95.96% live cells ± 2.89) and slightly lower for the B fabric (95.04% live cells ± 0.86), 1 cm for the B fabric (94.76% live cells ± 1.07), 1.5 cm for the NIKE fabric (94.60% live cells ± 1.17), slightly smaller for B (94.20% live cells ± 1.09), and 2 cm for B (93.66% live cells ± 1.42). Based on these results, it can be concluded that the B-type flax variety fabric had the best effect on cell viability.

**Figure 1:**
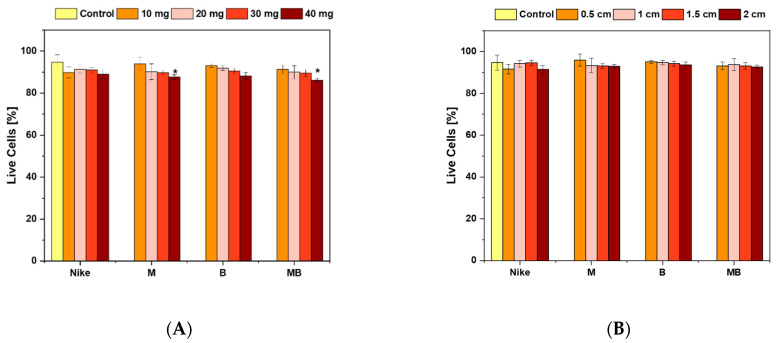
V79 cell viability after 48 h of incubation with fiber (**A**) and linen dressings (**B**) in the trypan blue cell staining assay (* *p* < 0.05).

When analyzing the cell survival rate only for the flax fibers alone, inconclusive results were obtained (Figure 1). The highest survival of V79 cells for the 10 mg sample of fiber was obtained for the M fiber (94.08% live cells ± 3.18) and for the 20 mg sample for the B fiber (92.00% live cells ± 1.18). In contrast, for samples above 30 mg, the highest cell survival was obtained for the NIKE fiber (30 mg—90.96% live cells ± 1.29; 40 mg—89.09% live cells ± 2.13), but these were lower values compared to the cell survival after treating V79 cells with fibers weighing 10 and 20 mg of the transgenic flax varieties. The greatest vitality was obtained for B.

**Figure 2:**
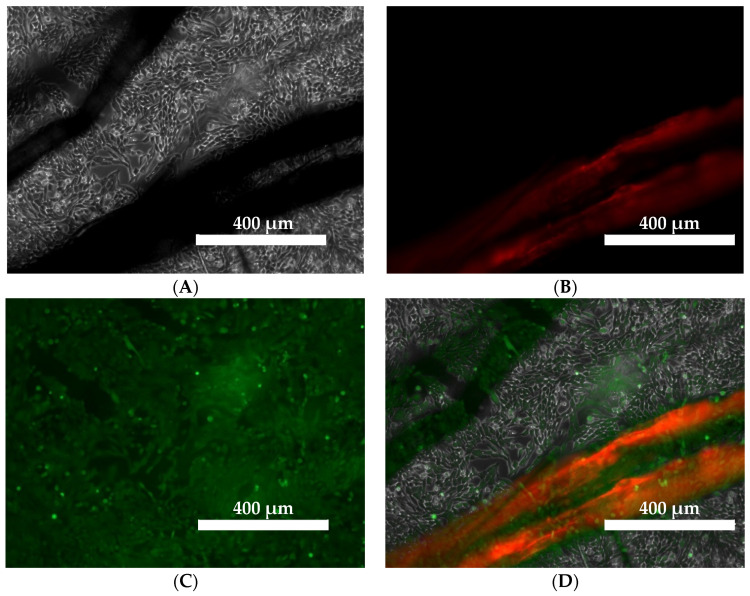
Microphotograph of a co-culture of V79 fibroblasts with linen fabric obtained in type-B plants. Cells were stained with the live dead cell staining kit (live cells fluoresce green and dead cells fluoresce red). Images were taken at 10x objective magnification with an EVOS FL microscope. (**A**). phase contrast, (**B**). GFP fluorescence, (**C**) RGB fluorescence, and (**D**) merging of three images.

Co-culture micrograph of V79 fibroblasts with linen fabric obtained from B-type plants. The number of living cells that fluoresce green predominates compared to dead cells with fluoresce red (Figure 2). The microphotograph also presented a red-dyed flax fiber. 

### 2.2. Cell Proliferation

**Figure 3:**
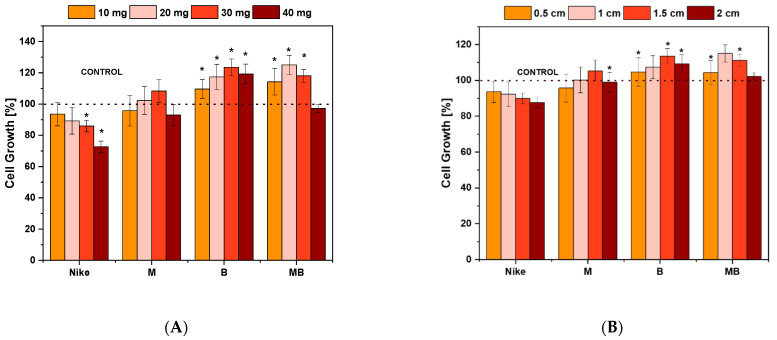
Cell proliferation of line V79 after 48 h of incubation with test flax fibers (**A**) and linen dressings (**B**) at four different concentrations and surfaces. The results represent means from 5 independent experiments ± SEM. Statistical significance of differences between results for test flax fabrics compared to the control (* *p* < 0.05).

The highest proliferation of V79 cells occurred for flax fibers compared to fabrics (Figure 3). All fabrics and flax fibers from the M, B, and MB-modified flax varieties cause greater cell proliferation than the control NIKE flax fabric and fiber. In the case of linen fabrics, B-flax (0.5 cm—104.66 cell grown ± 7.94, 1.5 cm—113.54 cell grown ± 4.26, and 2 cm—109.29 cell grown ± 5.03) causes the greatest cell proliferation. For 1 cm, the highest cell proliferation occurs for MB flax fabric (115.06 cells grown ± 4.75). For flax fibers, the highest proliferation values of 10 mg (114.27 cells grown ± 8.47) and 20 mg (125.06 cells grown ± 5.94) are found for MB fibers. In contrast, the highest proliferation appeared for B fiber for 30 mg (123.54 cells grown ± 5.33) and 40 mg (119.29 cell grown ± 6.29) fibers. However, at 40 mg fiber weight, V79 cell proliferation significantly decreased for all fibers. The highest values of cell proliferation were obtained for the MB fiber and fabric.

### 2.3. Evaluation of the Intracellular Free Radical Level

The amount of free radicals increases with the increased cell proliferation due to improved metabolic processes. The highest values of the level of free radicals after placing flax fibers and fabrics on V79 cells occurred for fabrics. The highest values of free radicals were found for linen fabrics with a size of 0.5 cm. At the same time, the fabric made of unmodified NIKE linen (9791.49 FAU ± 1712.35) achieved the highest amount of free radicals. Among the modified linen fabrics, the highest reactive oxygen species (ROS) values occurred for the linen fabric M (7845.15 FAU ± 2020.14).

**Figure 4:**
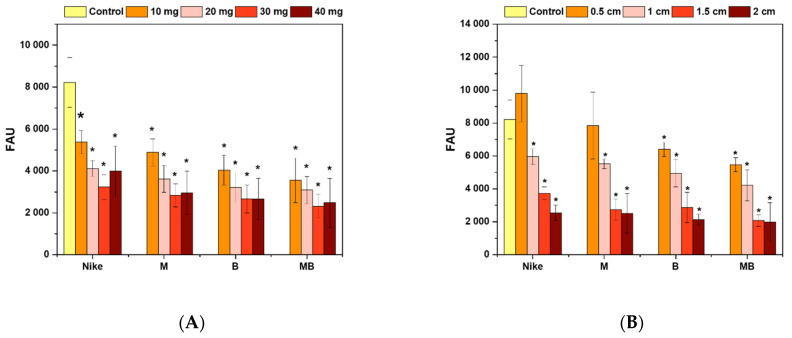
Influence of V79 cell incubation with the tested flax fiber (**A**) and linen dressing (**B**) on the levels of intracellular ROS induced by exposure to H_2_O_2_, as assessed with the 2′7′-dichlorofluorescein diacetate assay. Cell cultures were incubated for 48 h with flax fiber and linen dressing and then exposed to 100 µM H_2_O_2_. Control cultures were exposed to H_2_O_2_ without previous incubation with flax materials. Yhe fluorescence was read with a microspectrofluorimeter (λ ex = 485 nm, λ em = 535 nm). The results are presented as arbitrary fluorescence units (FAUs; mean ± SD, *n* = 5). The results (mean ± SD, *n* = 5) were compared to those for the control cultures, and statistically significant changes are marked with asterisks (* *p* < 0.05).

On the other hand, for fibers, the highest level of free radicals was also demonstrated for NIKE fibers with a sample of 10 mg (5370.82 FAU ± 555.25), and for modified fibers, it was for M (4885.08 FAU ± 648.35). The largest metabolic changes occur in NIKE fibers and fabrics and, to a lesser extent, for M-type fibers and fabrics. All tested flax fibers and fabrics reduced the level of free radicals compared to the control, which was treated with 100 μM H_2_O_2_ (Figure 4).

### 2.4. Genotoxicity Assessment

**Figure 5:**
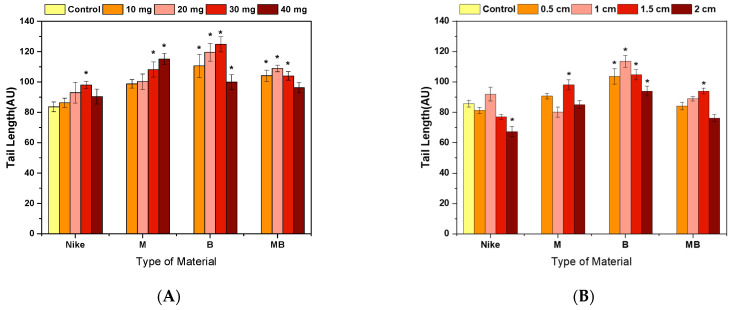
Effect of incubation of fiber (**A**) and linen dressings (**B**) on the amount of DNA damage measured by the length of the comet tail in the comet assay (* *p* = 0.05).

**Figure 6:**
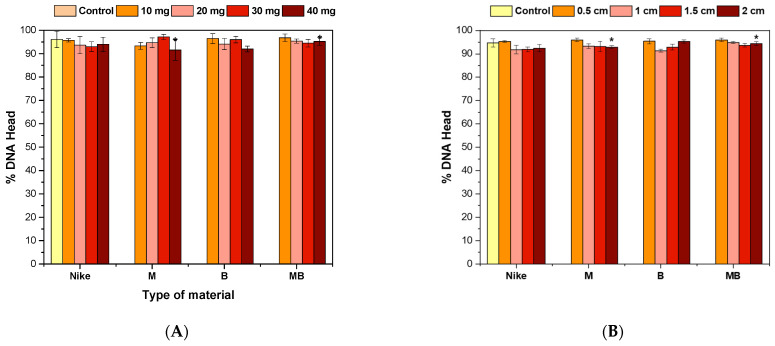
Effect of fiber (**A**) and linen dressing (**B**) incubation on the amount of DNA damage measured by the DNA content in the comet head in the comet assay (* *p* = 0.05).

**Figure 7:**
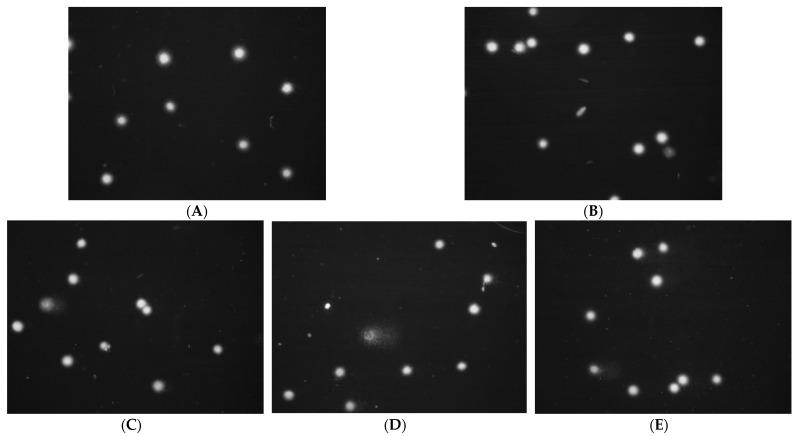
Effect of linseed dressings on the amount of DNA damage in cells in the comet assay. Lens magnification 20×, DNA staining with DAPI dye. (**A**) Control H_2_O_2_ 100 µM, 20 min, 4 °C; (**B**) NIKE; (**C**) M; (**D**) B; and (**E**) MB.

In the comet test for flax fibers and fabrics, an increase in the length of the tail is visible (Figure 5, Figure 6 and Figure 7). A visible haze near the comet indicates the following apoptotic process. There is no reduction in the amount of DNA in the comet’s head for fibers and fabrics. The comet head is a characteristic place where the test cells are located before lysis is carried out. The tails appear only when the cell is affected by a factor causing the degradation of the genetic material. The increase in tail length is caused by a small amount of badly damaged cells, which leads to apoptosis. The greatest increase in the tail length in the comet test was for flax fibers compared to flax fabrics. The longest tail was for the B fiber for the 20 and 30 mg samples, and for the dressings, it was for the B fiber fabric (0.5–1.5 cm), where cells disintegrate as a result of apoptosis. The tail length was similar to the control for the fibers and fabrics of the MB-type linen.

### 2.5. Potential Wound Environment Response to Oxidative Stress

**Figure 8:**
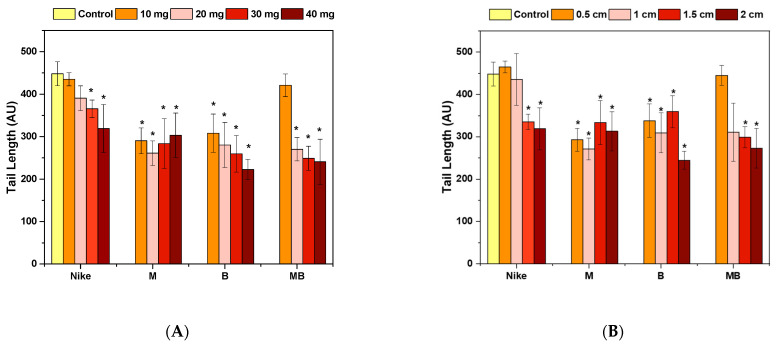
Protective effect of fiber (**A**) and linen dressing (**B**) incubation on the amount of DNA damage measured by the tail length in cells damaged with 100 µM H_2_O_2_ (20 min, 4 °C) in the comet assay (* *p* = 0.05). Control cells were incubated only with the damaging agent.

**Figure 9:**
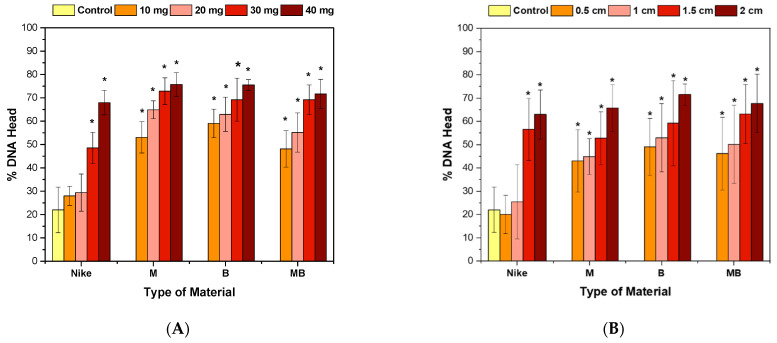
Protective effect of incubation with fiber (**A**) and linen dressings (**B**) on the amount of DNA damage measured by the DNA content in the nuclei of cells damaged with 100 µM H_2_O_2_ (20 min, 4 °C) in the comet assay (* *p* = 0.05). Control cells were incubated only with the damage agent.

The culture exposed to H_2_O_2_ illustrates the linen dressing behavior in the wound environment, where DNA (ssb) damage is exacerbated by the action of H_2_O_2_. The M linen fabric presented the best properties—thanks to which, the amount of damaged DNA was significantly reduced. In the case of the remaining fabrics, there is no deterioration of the DNA damage. Comparing the influence of flax and flax fibers on the potential wound environment, it turns out that flax fabrics definitely have a clear advantage in this analysis over flax fibers (Figure 8 and Figure 9). Of the fibers, the M and B fibers had the best properties in this test.

**Figure 10:**
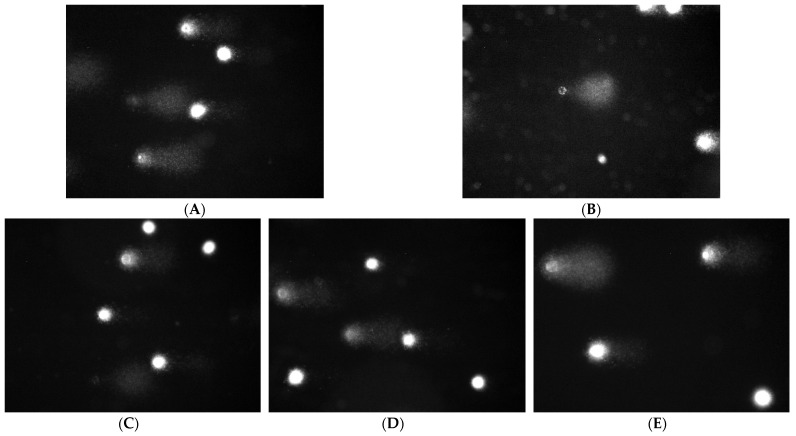
Protective effect of linen dressings on the amount of DNA damage to cells damaged by 100 µM H_2_O_2_ (20 min, 4C) in the comet assay. Magnification 20×, DNA staining with DAPI dye. (**A**) Control H_2_O_2_ 100 µM, 20 min, 4 °C; (**B**) NIKE; (**C**) M; (**D**) B; and (**E**) MB.

The V79 cell culture exposed to H_2_O_2_ (without preincubation with flax fabrics), it demonstrated severe damage on all nucleoid (comets) undamaged DNA, but when it used linen dressing M, there was less damage (Figure 10).

### 2.6. Apoptotic and Necrotic Cells

**Figure 11:**
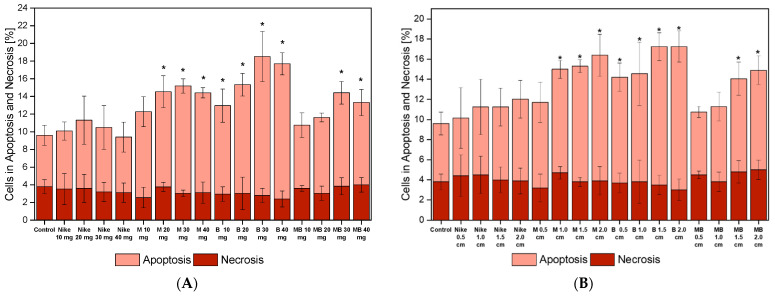
Apoptosis and necrosis of the V79 cells after 48 h of incubation with the tested flax fiber (**A**) and linen dressing (**B**) on four different surfaces. The results are presented as the percentage of apoptotic cells. The results are the means of 5 independent experiments. The statistical significance of the differences between the results for the tested linen fabric is compared to the control (* *p* < 0.05).

Fluorescein isothiocyanate (FITC) staining with annexin and propidium iodide was performed to assess the number of apoptotic and necrotic cells. The vast majority of cells for all flax fibers and fabrics were in the state of apoptosis. The distribution of necrosis and apoptosis was similar for flax fibers and dressings. Still, flax fabrics achieved higher apoptosis rates (Figure 11). The fewest cells in the apoptotic phase were observed in cultures with traditional linen fabric and the largest with linen fabric B.

### 2.7. Cell Cycle

**Figure 12:**
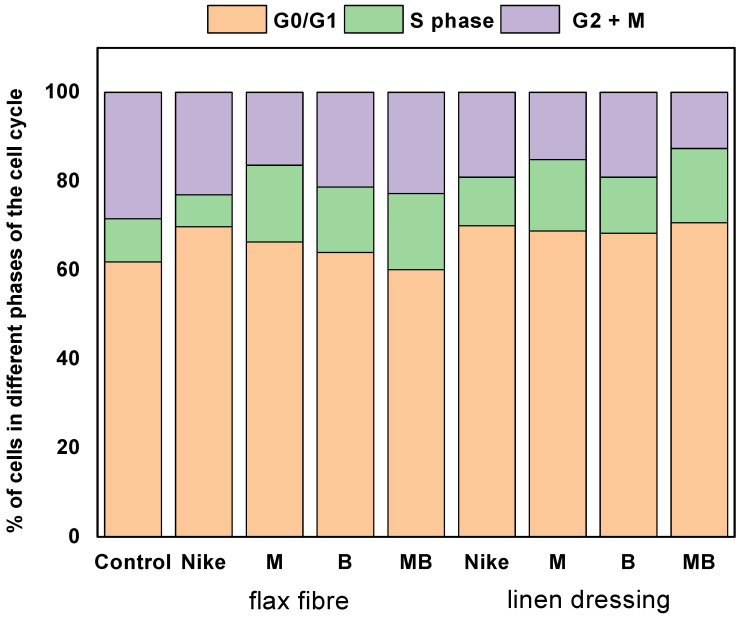
Cell cycle in V79 cells after 48-h incubation with the tested flax fibers and linen dressing. The results are presented as the percentage of cells in each phase. The results are the means of 5 independent experiments.

A cell cycle analysis was performed to see if incubation of V79 cells with the test fibers and fabrics increased the number of cells in the proliferative phase (S phase) after 48-h incubation (Figure 12). For all fibers and fabrics from transgenic flax varieties, there were more cells in the S phase compared to the fibers and fabrics from traditional NIKE flax and an increase in the number of cells in the G_0_/G_1_ phase. For fibers and fabrics from transgenic variants, a similar number of cells is observed in the S phase, while the number of cells is different for the G_0_/G_1_ and G_2_+M phases. For fibers of transgenic varieties of flax, the greatest number of cells in the G_0_/G_1_ phase occurs for the M fiber and the least for the MB fiber. In turn, the greatest number of cells in the G_2_+M phase occurs for the MB fiber. On the other hand, for fabrics made of transgenic flax, the opposite is true. The greatest number of cells in the G_0_/G_1_ phase occurs for the MB linen fabric and the least for the M and B linen fabrics. The smallest number of cells in the G_2_+M phase occurs for the MB linen fabric and the most for the B linen fabric. The G_1_ phase in the cell, even before the S phase of the cycle, in which the duplication of abnormal genetic information may occur, occurs when various mutations and DNA damage appear. There may also be a cell cycle arrest in the G_2_ phase, which prevents the formation of two defective daughter cells in the following M phase, hence the mitotic division phase. The obtained results indicate a favorable influence of the technological process of fiber on the obtained linen fabrics. Most preferably, it is directed to the programmed death of damaged cells at two cell cycle checkpoints, especially in the G_2_ and M phases.

### 2.8. Scratch Assay

**Figure 13:**
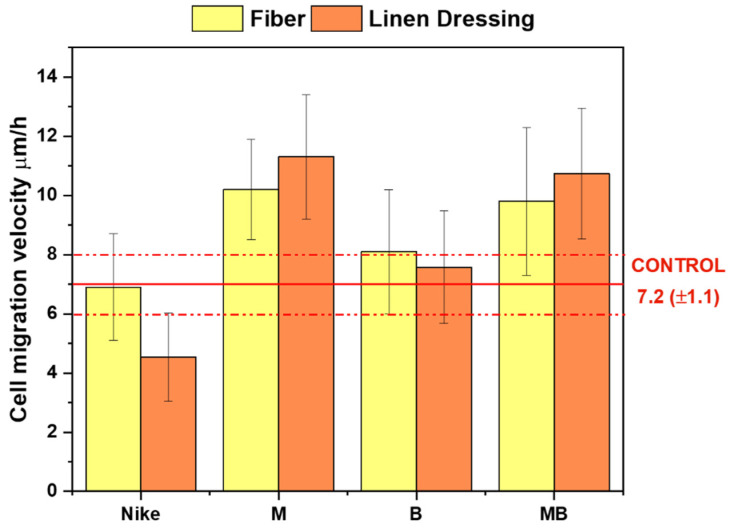
Average migration velocity of V79 lineage cells in um/h calculated after 20 h for flax fiber at a concentration of 20 mg/mL and a 1 cm^2^ flax dressing compared to the control.

A scratch test was performed to assess the cell migration and, thus, wound-healing potential (Figure 13). As expected, the degree of soiling was presented to be slower when using flax fibers of the test compounds compared to flax fabrics. All the tested fabrics from transgenic flax varieties resulted in faster cell migration than the NIKE linen fabric. M linen fabric indicated the strongest cell migration, with MB slightly smaller. A similar distribution of results was obtained for the flax fibers. The strongest cell migration occurred for the M fiber and slightly weaker for the MB fiber.

### 2.9. The Effect of Linen Fiber and Linen Dressing on Wound Healing in the V79 Cell Model

**Figure 14:**
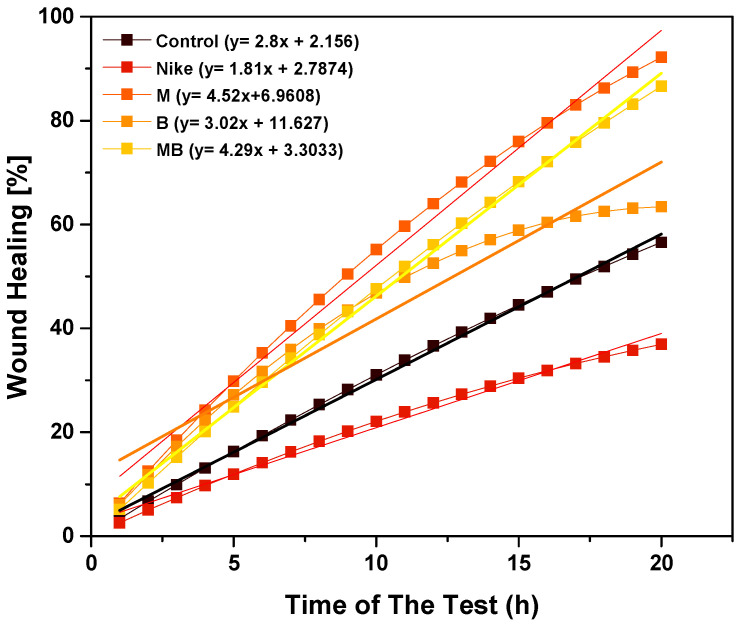
The effect of linen cloth on wound healing in a V79 cell model by assessing the increase in the confluence of the culture area at the injury site. The test was performed for a time of 20 h for linen fiber at a concentration of 20 mg/mL compared to the control.

**Figure 15:**
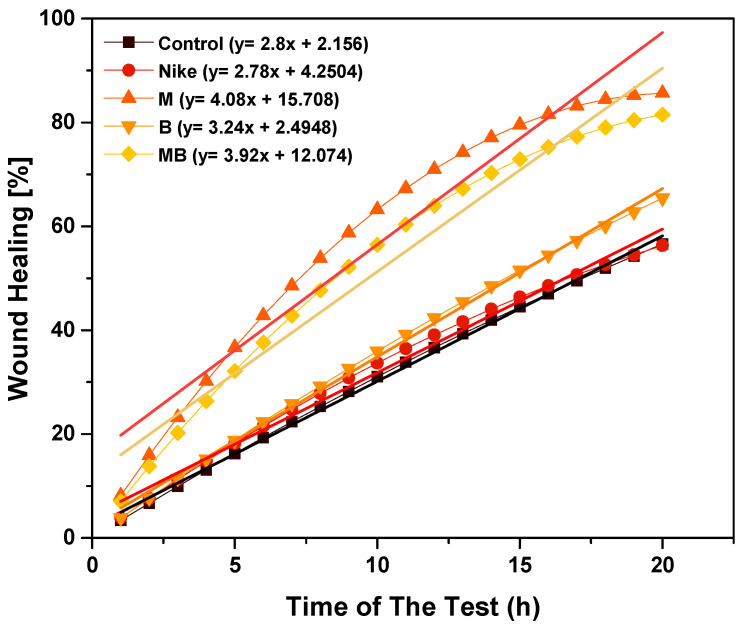
The effect of linen cloth on wound healing in a V79 cell model by assessing the increase in a confluence of the culture area at the site of injury. The test was performed for 20 h for a 1 cm^2^ linen dressing compared to the control.

The effect of fiber and linen dressing on wound healing was determined in the V79 cell model by assessing the increase in the confluence of the culture area at the site of the injury. The test was carried out for 20 h for flax fiber at a concentration of 20 mg/mL and for a 1 cm^2^ linen dressing compared to the control (Figure 14 and Figure 15). The most physiological course of the wound healing process is in the form of a logarithmic plot. In a V79 cell model for linen fabrics, such a course of the granulation process occurs for B and M linen fabrics. In contrast, the healing process is characterized by constant growth without the plateau phase for other fabrics. The healing process proceeds most favorably for the M fabric, as there is a plateau phase but with a higher percentage of the wound healed than the B fabric. Additionally, the plateau phase for the M fiber occurs at higher wound-healing percentages. The results obtained in the model present that the most favorable properties for wound healing are found for M flax fiber and fabric.

In the research carried out for this work, an attempt was made to determine the fiber and fabric with the best properties, summarized in Table 2. Furthermore, the correlation between apoptosis and the ROS assay and the genotoxicity test results is also determined in Table 3.

Based on the results of the tests performed, summarized in Table 2, it is difficult to select the fabric with the best properties unequivocally. However, it seems that the MB linen fabric presented the best properties.

Based on the presented results in Table 3, it is concluded that there is a strong correlation for the MB fiber and fabric, B fiber, and NIKE fabric between the level of free radicals and genotoxicity (the length of the tail) when the cell enters the apoptosis process and moderately strong for the NIKE fiber and B fabric. On the other hand, a moderately strong correlation between apoptosis and genotoxicity occurs for the NIKE and M fibers, and there is no correlation for the linen fabrics. The results indicate that the fiber strongly enhances damaged cell apoptosis. On the other hand, the dressing no longer exhibits such properties. There is a change in the properties between the fiber and the linen dressing, which indicates the influence of the technological process on the properties of the dressing, which induces more regenerative processes.

## 3. Discussion

Wounds have become one of the leading causes of death worldwide [20,21,22,23]. Chronic wounds are a problem for patients and the medical system in Poland and worldwide. Active or healed venous ulcers occur in 1% of the population, and pressure ulcers in 0.75% of the US [24,25]. In developed countries, up to 4% of the total expenditure is spent on treating chronic wounds [26]. A chronic wound is defined as a wound that does not heal within 3 months. The incidence of diabetes mellitus and other chronic diseases such as peripheral circulatory disorders and vascular diseases can impede wound healing [27,28,29,30]. Chronic wounds refer to damage to skin tissues caused by various causes. The healing process takes a long time, e.g., deep ulcers, including those formed after chemotherapy and radiotherapy, and in the course of a diabetic foot, as well as third to fourth-degree pressure ulcers [31]. With the aging of the population and the increasing number of comorbidities in the elderly, the problem of chronic wounds is increasing, and it is necessary to continue searching for new dressings with better healing properties [32,33].

However, the problem of difficult-to-heal wounds did not arise today. It has been accompanying people for millennia and is the subject of the search for effective methods of their treatment. Many natural methods have proved effective and have been used in natural medicine. Flax is an example of a natural raw material already used in antiquity [16]. The research subject was to compare the starting material, flax fiber, with the obtained dressing. Unfortunately, the industrial processing of even the best natural raw material often causes it to lose its health-promoting properties. Until now, unpublished studies presented that a linen dressing subjected to advanced industrial treatment, consisting of bleaching and improving the properties of the fabric itself by making it more elastic, caused its toxic effect on cell cultures. In the course of the research, the influence of the basic treatment of flax was checked in the context of its properties related to the wound-healing process.

The two main factors that are commonly thought of as common problems underlying nonhealing wounds are infection and wound inflammation. The hostile environment for wound healing creates inflammation. On the other hand, a difficult and slow-healing wound is more prone to infection. In turn, when a condition occurs, the inflammation of the wound is exacerbated [27,34]. However, wound healing is a complex process. In the first step, inflammation is induced, and the wound is debrided by finally removing dead cells from the wound. There will also be intense cell proliferation soon to restore the integrity of the damaged tissue. At the same time, a scarring process takes place in which the granulation tissue in the cell becomes fibrotic and hardened. At the same time, the excessive proliferation of fibroblasts may lead to excessive scarring and an impaired blood supply to the newly formed tissue [16,35,36].

During the application of the flax base, due to its properties, in the wet phase, due to the maintenance of optimal humidity and high hygroscopicity, flax slices can absorb exudate, which, in turn, significantly reduces the risk of infection and secondary infections. However, the patches have anti-inflammatory properties due to the enrichment of flax fibers with high concentrations of antioxidants, such as phenolic acids, vanillin, acetowanilone, and flavonoids. In contrast, the antioxidants are washed out of the wet dressing. Linen dressings support the natural stages of wound healing in all stages of healing.

Based on the conducted research, it was shown that the technological process of preparing linen dressings has a positive effect on their regenerative properties. It was observed that flax fibers induced a greater proliferation of V79 cells. However, a significantly increased proliferation of flax fiber mass (40 mg) already inhibited cell proliferation. The induction of cell proliferation in wound healing is indicated to restore the integrity of damaged tissue. However, the induction of cell proliferation in the wound healing process should occur after the first healing stage, which involves removing damaged cells from the wound. Initiating an intensive proliferation process too early may pose the risk of a faulty healing process. At the same time, the excessive proliferation process may lead to excessive scarring and impaired blood supply to the newly formed tissue. The wound-healing process is most favorable in the tested V79 cell model for the fabric of modified flax M, because the plateau phase is present. Therefore, dressings made of modified flax varieties are safe at every stage of wound healing and do not pose a risk of dangerous scarring. This work and the previous publication [16] showed that the fibers significantly increase the amount of total white in the cell cycle, which is only beneficial during the initial stage of wound healing. At the same time, the intensification of this process in the later stages is not advisable. At the same time, flax fibers are characterized by the ability to eliminate damaged cells. This is indicated by the presented studies on the apoptosis and necrosis of V79 cells. The fibers strongly induced apoptosis in injured V79 cells.

The flax dressings did not present any such properties. However, the linen dressings had stronger properties of controlled wound healing. This was indicated by the scratch tests performed, where modified linen fabrics intensified the migration of V79 cells on the scratch to a much greater extent than the fibers. The strongest migration of V79 cells was caused by a dressing made of M. According to the conducted research, the mechanism of wound healing of fibers and linen dressings is two-way, consisting of proapoptotic action of damaged cells and antioxidant activity. Flax fibers have a stronger proapoptotic effect compared to dressings, while linen dressings show stronger antioxidant properties and, at the same time, create a more favorable wound-healing environment. Linen dressings made of modified flax fibers induced a significant reduction in the number of free radicals, which allowed for the enhanced repair of DNA damage. This effect was observed for the M flax dressing. However, the remaining flax dressings did not increase the DNA damage in the cells.

Ideal wound dressings should have properties such as supporting tissue regeneration, enhancing cell proliferation and migration, and a biocombination. In addition, if there is wound exudate, it should be systematically removed from the wound environment by dressing [37,38]. Linen dressings made of modified flax varieties exhibit such properties.

The results indicate a strong fiber and linen dressing biological activity. The presented results indicate several mechanisms of action of the tested dressings. The tests confirmed the lack of toxicity for the cells of the V79 fibroblast line. Additionally, an acceleration of the cell growth was observed. The obtained results confirmed the results obtained in previous studies. 

Research in a series of publications on linen dressings with chronic wound-healing properties indicated that all the obtained linen dressings did not lose their healing properties under technological processes. In contrast, none of the linen fabrics tested were cytotoxic for the NHDF, HMCEV, and THP-1 fibroblast cultures. What is more, the tested fabrics caused a significant decrease in the total protein content in skin cancer [17]. Moreover, the tested fabrics resulted in a statistically significant decrease in the total protein content in skin cancer [17]. During the technological processes, no chemicals are used that may have a negative effect in contact with the wound and may reduce the bactericidal properties of the fibers. Compared to the traditional flax fiber, genetically modified M, B, and MB flax fibers had a stronger effect on the proliferation activity of keratinocytes, fibroblasts, and microvascular endothelium [16]. 

The key advantage of linen fabric is its mechanical properties as a dressing and biological properties related to the substances contained in linen. This action manifests itself in reducing free radicals and protecting DNA against the effects of oxidative stress. 

Skórkowska-Telichowska et al. presented the effects of woven fabrics from fibers derived from two types of transgenic plants were investigated: M-type plants, which produce the hydroxybutyrate polymer in their vascular bundles, and W92, which overproduce flavonoids. It was found that the incubation of V79 cells with these flax fabrics prevents ROS-induced chromatin instability and thus reduces DNA breakdown in the cells. The properties of the M fabric, which produces polyhydroxybutyrate, can also be compared to other linen fabrics from the transgenic plant W92, which overproduce phenylpropanoid compounds and are a source of antioxidants [13]. Wound healing is significantly improved by linseed dressings from plants containing more polyphenolic compounds [14]. Furthermore, the extracts containing phytosterols, cannabidiol (CBD), and flax fiber unsaturated fatty acids inhibited chronic inflammation and induced wound healing [39].

Similar effects were also obtained in patients during the clinical tests performed for the tested linen dressings. A 12-week pilot study of leg ulcer healing was also conducted by using a linseed dressing alone or in combination with seed extract and oil emulsion. Linen dressings show microbiological purity, are hypoallergenic, reduce the exudate and size of the wound, accelerate the healing process, and reduce the pain of the wound [11]. However, clinical trials have shown that they can be used on a diabetic foot with open varicose veins; severe thermal, chemical, and electrical burns; oozing wounds; bedsores; and mechanical damage to the body. In addition, they show very strong hygroscopic properties—they absorb exudate (deep, oozing wounds) and cleanse the tissue.

**Figure 16:**
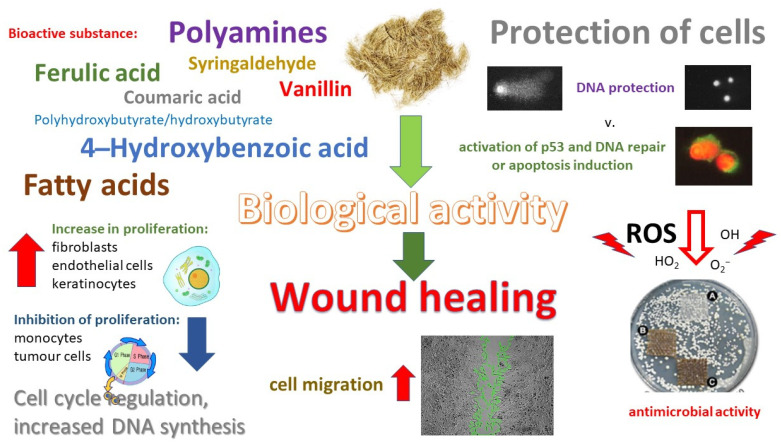
Mechanism of action of linen dressings (the microbiological evaluation was carried out by Kulma et al. [46]).

The mechanism of the action of linen dressings in wound healing is presented in Figure 16. The results of the in vitro and in vivo tests carried out so far, shown in Table 1, presented that the dressings obtained from the modified flax varieties do not indicate cytotoxicity in relation to all the cells and do not induce the progression of neoplastic cells. Moreover, linen dressings accelerate healing and reduce the exudation and wound size. Research conducted in 2017 presented that B and MB fabrics are the strongest activators of NHDF and are the most recommended dressings. On the other hand, the results of the research conducted on the V79 line so far show that the best properties are characteristic for dressings made of fabrics derived from modified MB flax. Moreover, the fibers are characterized by the ability to eliminate damaged cells in the elimination phase. However, the obtained fabrics acquire other properties during the technological processing of fibers into linen dressings. For example, linen fabrics have better regenerative properties for cells than fibers. Maintaining a sterile and moist wound microenvironment is a basic requirement for effective wound healing [40]. The current trends in dressing design focus on moistening the dressing with a linen oil emulsion, which should result in faster wound healing and additional antiviral properties. Natural phenolic acids are bactericidal and bacteriostatic [40,41,42,43,44]. Due to their properties, many metal nanoparticles, especially silver, have been used in medicine. Materials intended to be encapsulated into nanoparticles for medical applications must be nontoxic, chemically stable under various conditions, and biocompatible [45]. In further works, we want to focus on research on linen dressings containing silver nanoparticles, which will additionally increase the valence and use of linen dressings while extending the spectrum of their actions to dressings for infected wounds. 

## 4. Materials and Methods

### 4.1. Plant Materials

The research used flax fibers and linen fabrics obtained from them, which come from the traditional variety of linen (NIKE) and transgenic types of linens (M50 and B14) and their combinations (M50 + B14) [16,47]. The transgenic plants are from NIKE. B14 plants defensively transformed the potato β-1,3-glucanase (PR-2) gene [5,17,48]. M50 plants were enriched with Ralstonia eutropha genes encoding acetoacetyl CoA reductase (phbB), β-ketothiolase (phbA), and PHB synthase (phbC) for poly-β-hydroxybutyrate (PHB) [16,17,49]. A combination of these two flax fibers was M50 + B14 [16,17,47].

**Figure 17:**
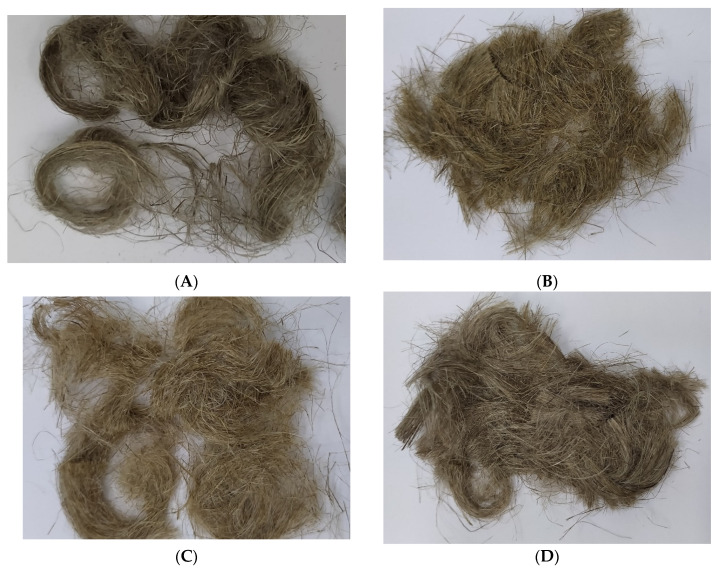
Flax fibers tested in the study: (**A**) NIKE plant fiber, (**B**) M plant fiber, (**C**) B plant fiber, and (**D**) MB plant fiber.

**Figure 18:**
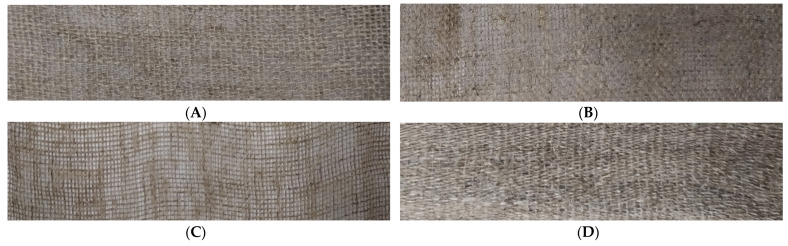
Linen fabrics: (**A**) linen fabric from NIKE flax, (**B**) linen fabric from M flax, (**C**) linen fabric from B flax, and (**D**) linen fabric from MB flax.

The diagram presented pictures of flax fibers and the linen fabrics obtained from them, serving as dressings, which were used to conduct the experiments in this work (Figure 17 and Figure 18).

### 4.2. Reagents

Eagle’s minimal essential medium (EMEM), fetal bovine serum (FBS), and trypsin/EDTA solution were obtained from Biological Industries (Beit-Haemek, Israel). The solution of antibiotics containing 10,000 U/mL penicillin, 10,000 μg/mL streptomycin, and 29.2 mg/mL L-glutamine (100×) was from Biological Industries (Beit-Haemek, Israel). Sulforhodamine B (SRB), 2,7′- dichlorodihydrofluorescein diacetate (DCFH-DA), 4′,6-diamidino-2-phenylindole (DAPI), dimethyl sulfoxide (DMSO), Trizma base, HEPES, Triton X-100, low melting point agarose (Sigma type VII), and regular agarose (Sigma type I-A) were purchased from Sigma-Aldrich (St. Louis, MO, USA). A fluorochrome mixture for detecting apoptosis (Alexa Fluor 488 Annexin V/Dead Cell Apoptosis Kit) was purchased from Invitrogen/Molecular Probes (Carlsbad, CA, USA). Phosphate-buffered saline (PBS), 0.4% trypan blue solution, NaOH, NaCl, and hydrogen peroxide (H_2_O_2_; 30% solution in water) were obtained from POCH (Gliwice, Poland). Plastic 75-cm^2^ growth area culture flasks and 24-well tissue culture-treated polystyrene culture plates for adherent cells were from SPL Life Sciences (Pochon, Korea). Disposable plastic pipettes and centrifuge tubes were from SPL Life Sciences (Korea).

### 4.3. Preparation of the Flax Fabric for Biological Tests

**Figure 19:**
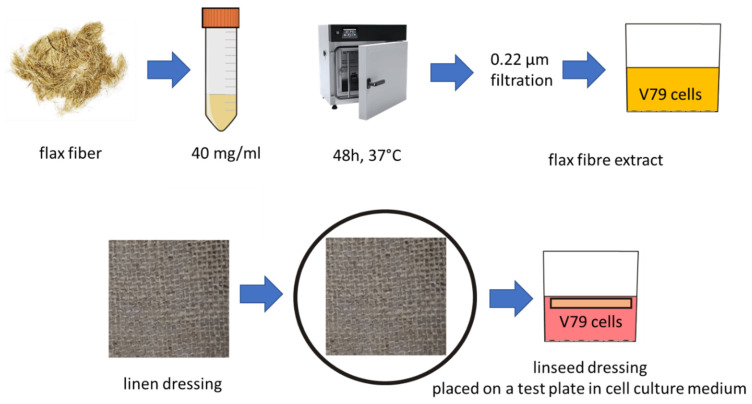
The process of preparing linen fibers for biological testing.

Four types of flax fibers (NIKE, M, B, and MB) were prepared and mixtures of 10–40 mg/mL in the culture medium. The fiber and culture medium mixture was then incubated for 48 h. Then, it was filtered. The extract obtained was added to the cell cultures for 48 h. The fibers were soaked in PBS, and the filtrate was collected for testing in the V79 cell culture. Linen fabrics were made out of four flax fibers (NIKE, M, B, and MB). The tested linen dressings were put into the culture medium (without PBS) for 10 min and placed in cell cultures on culture plates. The linen fabrics floated just below the surface of the growing medium, covered with a layer of substrate approximately 1 mm thick. The entire process of preparing linen fibers and fabrics for testing is presented in Figure 19.

### 4.4. Cell Line and Cell Culture Conditions

Chinese hamster pulmonary fibroblasts (V79–379A cells) were obtained from ATCC (USA). Cells were grown at 37 °C in a CO_2_ incubator in EMEM with 2 mM L-glutamine, 10% FBS, and a mixture of antibiotics: (0.1 mg/mL streptomycin and 100 U/mL penicillin). V79 cells were grown at 37 °C in a CO_2_ incubator. Adherent cells were detached from culture plates with trypsin/EDTA solution, washed with PBS, spun out, counted, stained with a 0.4% solution of trypan blue, and inspected under a microscope for cell viability. The cells were plated on 1.5 × 10^5^ cells per well in 24-well plates and 5 × 10^4^ cells per well in 96-well plates. They were incubated for 24 h at 37 °C in a CO_2_ incubator for cell adaptation after the reseeding procedure. The flax fabrics and linen dressing were added to the cell cultures, and the cultures were placed in a CO_2_ incubator at 37 °C for 48 h.

### 4.5. Cell Viability

The influence of linen fibers and fabrics on vitality was assessed using the V79 cell line. After 24-h treatment, cells with tested fibers and fabrics were rinsed with PBS, which was collected into prepared centrifuge tubes. The culture supernatant was collected. Culture plates were washed with the TrypLe solution. The TrypLe solution was readded and culture plates were incubated for 2 min at 37 ° C. The solution with cells was collected and centrifuged at 600× *g* for 5 min. After the supernatant removal, cells were resuspended in PBS, and propidium iodide was added. After 5 min of incubation, the dark samples were analyzed on an Arthur image cytometer. In addition, live cell staining was performed using the LIVE/DEAD™ Cell Imaging Kit (488/570) (Life Technologies, Carlsbad, CA, USA). Stained cells were evaluated using an EVOS FL microscope (Thermo Fisher Scientific (Waltham, MA, USA) [50].

### 4.6. Cell Proliferation

The potential of the cells in the V79 line was assessed using the sulforhodamine B (SRB) test. Cell cultures with test fibers and fabrics were fixed with cold trichloroacetic acid (TCA) at 4–8 °C for 30 min after 48 h of incubation. The plates were washed five times under running water. After drying, the dye sulforhodamine B was added for 30 min. Unbound dye was removed through five rinses with 1% acetic acid and dried. Finally, the protein was dissolved in Trisma solution, and the absorbance at 555 nm was measured using a microplate reader (Victor2, PerkinElmer, Waltham, MA, USA).

### 4.7. Evaluation of the Intracellular Free Radical Level

According to the procedure [51], 2′7′-dichlorodihydrofluorescein diacetate (DCFH-DA) in a concentration of 25 μM was added for the last 2 h of culture and the V79 cells. It was incubated in the dark in the CO_2_ incubator. Afterward, the cells were washed twice with PBS, and H_2_O_2_ (100 μM) was added to each culture for 30 min. The fluorescence of dichlorodihydrofluorescein was then read (λex = 485 nm, λem = 535 nm) with a Victor 2 microspectrophotometer (PerkinElmer, Waltham, MA, USA).

### 4.8. Comet Assay

Alkaline single-cell gel electrophoresis (comet test) was performed according to the procedure [52]. The cells were cultured in the presence of flax fibers and fabrics for 48 h. V79 cells were separated with trypsin/EDTA from the culture vessel, centrifuged, and washed in PBS chilled to 4 °C without Ca^2+^ or Mg^2+^ ions. Cells were incubated with PBS supplemented with H_2_O_2_ (100 µM) in an ice-water bath (4 °C) for 30 min. Incubation was terminated by dissolving the cells with an excess volume of chilled PBS, centrifugation, and resuspending the cell pellet in cold PBS containing Ca^2+^ and Mg^2+^ ions. The suspension cells were mixed with an equal volume of 1% low melting point agarose (Sigma VII) prewarmed in a 37 °C water bath. The suspensions were then placed on slides precoated with 0.5% plain agarose (Sigma I-A type). Coverslips were removed and slides kept carefully immersed in cold (4 °C) lysis solution (2.5M NaCl, 100 mM EDTA, 10 mM Tris, pH 10, 1% Triton X-100, and 10% DMSO) and held overnight. in the dark at 4 °C. The slides were then washed (five times for 5 min each) with alkaline electrophoresis buffer (300 mM NaOH and 1 mM EDTA, pH 13) and then placed in a horizontal gel electrophoresis unit filled with freshly prepared alkaline electrophoresis buffer. The slides were exposed to alkali for 45 min at 4 °C. Electrophoresis was performed (1.2 V/cm, 300 mA) for 20 min at 4–6 °C, and the slides were washed with neutralizing buffer (0.4 M Tris, pH 7.5) four times for 5 min. Finally, the slides were immersed in a fluorescent dye (DAPI, 1 µg/mL), covered with coverslips, and stained overnight in a refrigerator. All steps were performed in dim light. The slides were analyzed using a Nikon Eclipse E600 microscope in the Comet IV program.

### 4.9. Apoptotic and Necrotic Cells

V79 cells were separated from the culture plates, centrifuged, resuspended in binding buffer, and stained with a mixture of fluorochromes (Alexa Fluor 488 Annexin V and PI fluorescent dyes). After 15 min of incubation at room temperature in the dark, samples were taken on the IMAGE base cytometry Arthur cytometer. The fluorescence, granulation and size were measured for 40,000–10,000 cells. The percentage of viable, apoptotic, and necrotic (dead) cells was calculated from the scatter plots.

### 4.10. Cell Cycle

The V79 cell cycle was assessed after 24 h of treatment of the linen fibers and fabrics. After incubating, the cells were separated and centrifuged, and then, the pellet was fixed with cold ethanol (70%) for 10 min at room temperature and centrifuged again at 600× *g* for 5 min. The cell pellet was resuspended in propidium iodide solution and left in the dark for 10 min. Samples were transferred to chips and analyzed on an Arthur image-based cytometer (NanoEnTek Inc., Seoul, Korea).

### 4.11. Scratch Test

**Figure 20:**
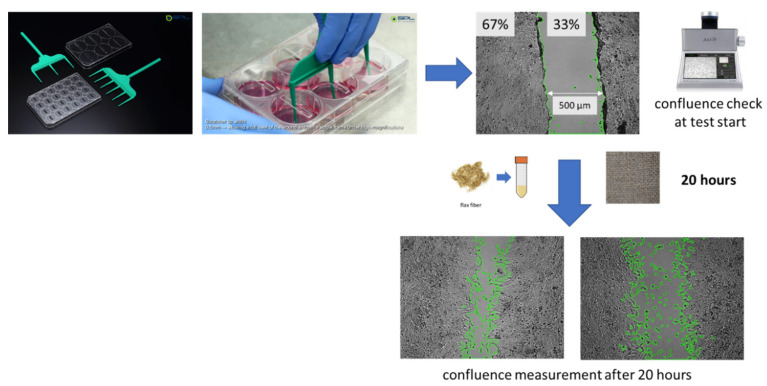
Scheme of the scratch test and microphotos.

After inoculating the cells, they were incubated until they formed a monolayer over the entire surface of the well. The SPLScar kit (SPL Life Sciences, Korea) was used to perform the scratch test. A scratch of the same thickness and in the same place in each well of the plate covered with cells was made using a scratcher. Pictures were taken. The prepared linen fibers and fabrics were added to the monolayers scratched on the surface. The culture plates were incubated for 24 h. The micrographs and their analyses were made using Julia’s microscope (Figure 20). The equipment took pictures showing the rate of fouling during 24 h of incubation in a CO_2_ incubator. The use of a dedicated test system allowed for high repeatability of the test. The obtained scratch area was 33% of the photographed area (±1%).

### 4.12. Statistical Analysis

All biological tests were performed in five independent replicates. Due to the normal distribution and equal variance of the obtained results, statistical calculations were performed with parametric tests. Using Statistica v.13 software, the statistical significance was calculated using Tukey’s post hoc test by using Statistica v.13 software. The significance point was set at * *p* < 0.05.

## 5. Conclusions

This conducted research confirms the knowledge known since antiquity that linen fabric has good properties that promote wound healing. The main aim of the research was to confirm that a very good raw material, which is linen fiber, does not lose its properties in the process of post-industrial processing. It is difficult to use the fiber directly in the treatment of wounds, so it was important to check the properties of the fabric obtained.Flax fabrics have the most favorable potential wound-healing properties compared to fibers. Better properties also characterize linen dressings made of modified varieties of linen compared to traditional linen fabric. Based on the tests performed, we indicated that the most advantageous properties have the dressings made of fabrics derived from modified MB flax. Linen dressings are made of woven fibers, which is why they help to cleanse wounds and ulcers and stimulate the growth of blood vessels. Thanks to the unsaturated fatty acids contained in the patches, the young tissue is strengthened and protected against drying out. The wound is protected against mechanical irritation or possible infection. In the epithelialization phase, linen dressings facilitate the epithelium’s growth by maintaining an appropriate moisture level and thus protect the tissue against damage. Linen dressings are still subject to research and development processes. The tested flax fibers are characterized by a greater ability to eliminate damaged cells in potential wounds. However, during technological processing, linen dressings acquire more regenerative properties for damaged cells. Works on the genetic improvement of flax and its use in medicine or other industrial areas are still ongoing. The effectiveness of flax dressings has been confirmed by studies and clinical tests that have presented a reduction or disappearance of wounds. Most importantly, they are not prone to induce allergies, and so far, no side effects have been observed.

The obtained results indicate a two-way action consisting, on the one hand, of enhancing the process of tissue regeneration and protection and, on the other hand, of removing damaged cells through apoptosis.

## Figures and Tables

**Table 1:** The results of the research on the influence of flax dressings.

Research Group	Material	Results
Skórkowska-Telichowska Katarzyna et al. [11]	Balb/3T3 cell line 30 patients with wounds that had lasted at least 2 years	- Modified linen dressings do not cause cytotoxicity and do not adversely affect the growth and morphology of Balb/3T3 cells. - After a 12-week application of a modified linen dressing, it follows accelerates healing and reduces exudate and wound size - Modified flax dressing reduced the pain associated with chronic venous ulceration.
Paladini et al. [12]	Balb/3T3 cell line	- The technology of silver nanophase deposition in flax dressings has proven to be effective and can be transferred from the laboratory scale to the macro scale - Linen dressings with nanosilver have antibacterial properties against G+ and G− be bacteria due to a loss of influence on cell viability.
Skórkowska-Telichowska Katarzyna et al. [13]	V79 cell line	- Linen fabrics from genetically modified flax W92 and M reduce DNA damage in V79 cells and produce fewer free radicals.
Gębarowski Tomasz et al. [14]	NHDF cell line	- Modified flax fiber and fabric extracts M, B, and M + B are not cytotoxic to NHDF cells and do not cause the growth of apoptotic cells in cell cultures. - Linseed dressings increase the proliferation of fibroblasts. - B and M + B flax dressings are the strongest activators of NHDF and are the most recommended dressings.
Gąsiorowski Kazimierz et al. [15]	NHEK, NHDF, HUVEC, THP-1 cell lines	- Dressings made of modified flax with an overexpression of phenolic acids and flavonoids (W92) and polyhydroxybutyrate (M48) release these substances from the cell culture dressings, giving better properties to the dressings. - Modified linen fabrics increase the proliferation of keratinocytes and fibroblasts.
Gębarowski Tomasz et al. [16]	Balb/3T3, NHDF, THP-1, NHEK, HMVEK, A431 cell lines	- Modified flax fibers increase the proliferation of fibroblasts and keratinocytes, reducing the number of free radicals. - Linen fibers do not stimulate the proliferation of cancer cells.
Skórkowska-Telichowska Katarztna et al. [5]	NHDF cell line 22 patients suffered from chronic non-healing ulcerations	- Modified flax dressing supports the removal of necrotic remnants from the wound, absorbs exudate, and provides an appropriate environment for the healing of exudative and infected wounds.
Gębarowski Tomasz et al. [17]	NHEK, Balb 3T3, HMCEV, THP-1 cell lines	- Linen dressings do not increase the number of necrotic cells. - Modified flax dressings reduce the amount of proteins in cancer cells. - All linen dressings did not lose their wound-healing properties under the influence of technological processes of processing flax fibers.

**Table 2:** The comparison of biological test results for flax fibers and fabrics.

Test	Fiber	Fabric
Cell viability	B	B
Cell proliferation	MB	MB
Free Radical Level	NIKE	NIKE
Genotoxic in the comet assay	MB	MB
Potential wound environment response to oxidative stress	M, B	MB
Apoptosis	B	B
Cell cycle	MB	MB
Scratch test—migration assay	M, MB	M, MB
The effect on wound healing in the V79 cell model	The first M, then MB	The first B, then M

**Table 3:** Designated correlations for apoptosis.

* DCFDA (H_2_O_2_)/** Tail (H_2_O_2_)				
	*** NIKE	**** M	**** B	***** MB
fiber	0.699	−0.084	0.892	0.879
fabric	0.938	−0.686	0.452	0.874
apoptosis/tail				
fiber	0.622095	0.678914	0.04192	−0.22859
fabric	−0.64786	0.00032	−0.68978	−0.17688

* DCFDA—Cellular ROS Assay/Reactive Oxygen Species Assay; ** tail—represents damaged DNA fragments in the comet assay; *** NIKE—fiber or linen without genetic modification; **** M and B—fiber or linen with genetic modification; and ***** MB—combination of M and B.

## Data Availability

Raw data can be obtained after e-mail contact with the corresponding author.
